# The need for comparative data in spondyloarthritis

**DOI:** 10.1186/s13075-019-1812-3

**Published:** 2019-01-22

**Authors:** Ernest Choy, Xenofon Baraliakos, Frank Behrens, Salvatore D’Angelo, Kurt de Vlam, Bruce W. Kirkham, Mikkel Østergaard, Georg A. Schett, Michael Rissler, Kamel Chaouche-Teyara, Chiara Perella

**Affiliations:** 1 CREATE Centre, Division of Infection and Immunity, Cardiff University School of Medicine, Wales, UK; 2 Rheumazentrum Ruhrgebiet, Ruhr-University Bochum, Herne, Germany; 3 CIRI/Rheumatology and Fraunhofer TMP, Goethe-University, Frankfurt, Germany; 4 Rheumatology Department of Lucania and Rheumatology Institute of Lucania (IRel), San Carlo Hospital of Potenza, Potenza, Italy; 5 Division of Rheumatology, University Hospitals Leuven, and Skeletal Biology and Engineering Research Center, Department of Development and Regeneration, KU Leuven, Leuven, Belgium; 6 Guy’s and St Thomas’ NHS Foundation Trust, London, UK; 7 Copenhagen Center for Arthritis Research (COPECARE), Center for Rheumatology and Spine Diseases, Rigshospitalet, Glostrup, and Department of Clinical Medicine, University of Copenhagen, Copenhagen, Denmark; 8 Friedrich-Alexander University Erlangen-Nurnberg and Universitätsklinikum Erlangen, Erlangen, Germany; 9 Novartis Pharma AG, Basel, Switzerland

**Keywords:** Biological therapy, Clinical trials, Psoriatic arthritis, Radiography, Spondyloarthropathy

## Abstract

Spondyloarthritis comprises a group of inflammatory diseases, characterised by inflammation within axial joints and/or peripheral arthritis, enthesitis and dactylitis. An increasing number of biologic treatments, including biosimilars, are available for the treatment of spondyloarthritis. Although there are a growing number of randomised controlled trials assessing treatments in spondyloarthritis, there is a paucity of data from head-to-head studies. Comparative data are required so that clinicians and payers have the level of evidence required to inform clinical decision-making and health economic assessments. In the absence of head-to-head studies, statistical methods such as network meta-analyses and matching-adjusted indirect comparisons (MAICs) are used for assessing comparative effectiveness.

Network meta-analysis can be used to compare treatments for trials using a common comparator (e.g. placebo); however, for those without a common comparator or where considerable heterogeneity exists between the study populations, a MAIC that controls for differences in study design and baseline patient characteristics may be used. MAICs, unlike network meta-analyses, are of value for longer-term comparisons beyond the placebo-controlled phase of clinical trials, which is important for chronic diseases requiring long-term treatment, like spondyloarthritis. At present, there are a number of limitations that restrict the effectiveness of MAIC, such as the poor availability of individual patient-level data from trials, which results in patient-level data from one trial being compared with published whole-population data from another. Despite these limitations, drug reimbursement agencies are increasingly accepting MAIC as a means of comparative effectiveness and greater methodological guidance is needed.

This report highlights a number of challenges that are specific to conducting comparative studies like MAIC in spondyloarthritis, including disease heterogeneity, the paucity of biomarkers and the duration of studies required for radiographic endpoints in this slow-progressing disease.

## Background

Spondyloarthritis (SpA) represents a group of inflammatory arthritides, mainly axial SpA and psoriatic arthritis (PsA), which share common genetic features and a chronic clinical course. There are a growing array of biologic agents and biosimilar medications, targeting key mediators involved in the inflammatory process, available to rheumatologists. Although there are an increasing number of randomised controlled trials (RCTs) assessing treatments in SpA, there is a paucity of data from head-to-head studies, so other forms of treatment comparison are needed. This commentary summarises the key findings of an industry-sponsored roundtable discussion involving eight expert rheumatologists and two medical personnel from the pharmaceutical industry, that was conducted in April 2017 to obtain advice and perspectives on comparative studies in SpA.

## Network meta-analyses and MAICs

Indirect comparisons can infer the relative effectiveness of different treatments. For trials using common comparators (e.g. Drug A vs Drug B and Drug B vs Drug C), treatments can be indirectly compared using a network meta-analysis [1]. Where there is no common comparator to facilitate a network meta-analysis, or where considerable heterogeneity exists between the study populations, a matching-adjusted indirect comparison (MAIC) that controls for differences in study design and baseline patient characteristics may be used [1, 2]. Significant debate exists regarding which indirect comparison method is optimal (Table 1) [3, 4].

**Table 1:** Advantages/disadvantages of NMA and MAIC

Network meta-analysis (NMA)	Matching-adjusted indirect comparison (MAIC)
Advantages	
• Compares multiple treatments using published aggregate data • Can connect head-to-head RCTs and other RCTs via a common comparator (usually placebo) • Multiple simultaneous indirect paths • Based on relative effects, so randomisation is preserved • Established methodology	• Reduces heterogeneity between trials by matching the patient population • Treatment effects have clear clinical context for interpretation • Possible with and without placebo adjustment • Long-term analyses feasible
Disadvantages	
• Assumes trials are comparable in terms of design and population (low heterogeneity) • Requires a common comparator (connected evidence network) • Often only short-term comparison due to lack of a long-term connected network (placebo switching)	• Evolving method—NICE Technical Support Document published in December 2016 [2] • Interferes with/breaks randomisation • Reduced patient sample size • Only a single indirect path • Can only match observed characteristics, so heterogeneity may remain

Adapted from Ishak et al. [1]

*MAIC* matching-adjusted indirect comparison, *NMA* network meta-analysis, *RCT* randomised controlled trials

Several MAICs have been published comparing biologics for the treatment of AS [5–12] and PsA [13–24], although only two as full-length papers [13, 25]; these analyses compare patient-level data from a trial of a given treatment (i.e. index trial) with published data for another treatment (Table 2) [1]. Until the National Institute of Health and Care Excellence (NICE) published technical guidance on this topic in December 2016 [2], the lack of quality criteria made it challenging to interpret the results of MAICs; however, further methodological guidance would be of value.

**Table 2:** Overview of published MAIC in SpA*

Year	Patient-level data	Published data	Sponsor	Type of publication
	Treatment A (trial name; ClinicalTrials.gov identifier)	Treatment B (trial name; ClinicalTrials.gov identifier)		
Ankylosing spondylitis				
2016	Secukinumab (MEASURE 2; NCT01649375)	Adalimumab (ATLAS; NCT00195819)	Novartis	Abstract [5]
2016	Adalimumab (ATLAS; NCT00195819)	Secukinumab (pooled MEASURE 1 [NCT01358175], MEASURE 2 [NCT01649375])	Abbvie	Abstract [6]
2016	Secukinumab (MEASURE 2; NCT01649375)	Adalimumab (ATLAS; NCT00195819)	Novartis	Abstract [7]
2016	Secukinumab (MEASURE 1; NCT01358175)	Adalimumab (ATLAS; NCT00195819)	Novartis	Abstract [8]
2016	Secukinumab (pooled MEASURE 1 [NCT01358175], MEASURE 2 [NCT01649375])	Adalimumab (ATLAS; NCT00195819)	Novartis	Abstract [9]
2017	Secukinumab (pooled FUTURE 1 [NCT01392326], FUTURE 2 [NCT01752634])	Golimumab (GO-RAISE; NCT00265083)	Novartis	Abstract [10]
2017	Secukinumab (pooled FUTURE 1 [NCT01392326], FUTURE 2 [NCT01752634])	Golimumab (GO-RAISE; NCT00265083)	Novartis	Abstract [11]
2017^a^	Secukinumab (MEASURE 2; NCT01649375)	Adalimumab (ATLAS; NCT00195819)	Novartis	Abstract [12]
Psoriatic arthritis				
2013	Adalimumab (ADEPT; NCT00195689)	Etanercept (Mease et al. 2004), infliximab (IMPACT 2; NCT00051623)	Abbvie	Manuscript [13]
2015	Adalimumab (ADEPT; NCT00195689)	Secukinumab (pooled FUTURE 1 [NCT01392326], FUTURE 2 [NCT01752634])	Abbvie	Abstract [14]
2016	Secukinumab (FUTURE 2; NCT01752634)	Adalimumab (ADEPT; NCT00195689)	Novartis	Abstract [15]
2016	Secukinumab (FUTURE 2; NCT01752634)	Adalimumab (ADEPT; NCT00195689)	Novartis	Abstract [16]
2016	Secukinumab (FUTURE 2; NCT01752634)	Etanercept (Mease et al. 2004)	Novartis	Abstract [17]
2016	Secukinumab (pooled FUTURE 1 [NCT01392326], FUTURE 2 [NCT01752634])	Adalimumab (ADEPT; NCT00195689)	Novartis	Abstract [18]
2016	Secukinumab (FUTURE 2; NCT01752634)	Infliximab (IMPACT 2; NCT00051623)	Novartis	Abstract [19]
2016^a^	Secukinumab (FUTURE 2; NCT01752634)	Adalimumab (ADEPT; NCT00195689)	Novartis	Abstract [20]
2017	Secukinumab (pooled FUTURE 1 [NCT01392326], FUTURE 2 [NCT01752634])	Interferon (IMPACT 2; NCT00051623)	Novartis	Abstract [21]
2017^a^	Secukinumab (FUTURE 2; NCT01752634)	Infliximab (IMPACT 2; NCT00051623)	Novartis	Abstract [22]
2017^a^	Secukinumab (FUTURE 2; NCT01752634)	Etanercept (Mease et al. 2004)	Novartis	Abstract [23]
2017^a^	Secukinumab (FUTURE 2; NCT01752634)	Adalimumab (ADEPT; NCT00195689)	Novartis	Abstract [24]
2017	Adalimumab (ADEPT; NCT00195689)	Secukinumab (pooled FUTURE 1 [NCT01392326], FUTURE 2 [NCT01752634])	Abbvie	Manuscript [25]

*Up to December 2017

^a^Cost per responder analysis

## Key points to consider for MAICs

First, making individual patient-level data available from trials will facilitate MAIC and circumvent the need to compare patient-level data from one trial with published whole-population data from another. Second, selection of baseline parameters for matching is critical. Ideally, these should be based on established key parameters that are known, from published data and expert opinion, to influence outcomes. Third, when placebo response is available, this can inform quality of matching. Additionally, anchored comparisons (i.e. having a common comparator) are preferred to unanchored analysis [26]. Fourth, if the number of patients included after matching is small, caution is needed when inferring results. Fifth, sensitivity analyses should be conducted, varying the matching parameters to assess the robustness of results or cross-validating the results using data from other trials. Lastly, a thorough and detailed description of the methodology is essential when reporting results.

## Challenges for MAICs in spondyloarthritis

In comparison to rheumatoid arthritis, SpA has a heterogeneous clinical presentation characterised by inflammation affecting the axial skeleton (the spine and sacroiliac joints), peripheral joints and entheses and extra-articular sites, such as the eye (uveitis), skin (psoriasis) and gut (inflammatory bowel disease [IBD]) [27]. This heterogeneity adds complexity when defining the inclusion criteria for a clinical study population and also for any subsequent MAIC that might only include a subset of the index population in order to match the comparator population. Treatment history is also important, as many newer studies include patients who have failed previous biologics. Information about the inclusion/exclusion criteria, recruitment processes and baseline characteristics of study populations are thus needed to inform indirect comparisons.

The complex and heterogeneous nature of SpA confounds the identification of a single representative outcome measure [28]. Although composite scores have been developed to assess disease activity in rheumatoid arthritis, the identification of a common composite measure for SpA conditions, including PsA, has proved more challenging due to the involvement of multiple organ systems and the variety of clinical presentations [28]. For axial SpA, including AS, the Ankylosing Spondylitis Disease Activity Score (ASDAS) is increasingly used, while in PsA, the use of different measures, such as the Composite Psoriatic Arthritis Disease Activity Index (CPDAI), the Psoriatic Arthritis Disease Activity Score (PASDAS), Minimal Disease Activity (MDA) and the Disease Activity Index for Psoriatic Arthritis (DAPSA), illustrates that no consensus has yet been reached. As there are limited data available for more recently developed outcomes (e.g. ASDAS), this restricts the utility of this outcome in MAICs. In addition, the heterogeneity of the individual disease manifestations included in composite scores may limit which score is compared in MAICs. For example, in ASDAS, C-reactive protein or erythrocyte sedimentation rate values are included, both of which may be affected by biologic disease-modifying antirheumatic drugs (DMARDs); however, neither of these are included in the Bath Ankylosing Spondylitis Disease Activity Index (BASDAI). Since not every patient with axial SpA will show a positive inflammatory finding in their laboratory examination or even fluctuation of these parameters, treatment with biologic DMARDs may affect ASDAS scores more in patients with inflammation than in those without [29]. Thus, certain composite scores may be more difficult to weigh the individual populations based on the heterogeneity of the individual disease manifestations.

Patient-reported outcomes (PROs) are important for consideration in health economic models for SpA owing to the considerable impact of PsA and AS on health-related quality of life. A recent systematic review identified 60 unique PRO measures for SpA, which differed in their degree of correlation with clinical variables [30]. The same study identified a lack of validation of PROs for use across SpA subtypes. For individual SpA subtypes, PROs used include the BASDAI and the Bath Ankylosing Spondylitis Functional Index (BASFI) in AS, while the Psoriatic Arthritis Quality of Life (PsAQoL) and Health Assessment Questionnaire Disability Index (HAQ-DI) are used in PsA. Little data is available on the use of these PROs in MAICs. In the two published MAICs in PsA, no statistically significant differences were observed between treatment arms in measurements of HAQ-DI, although differences in efficacy were noted in other more stringent endpoints, such as ACR and PASI response rates [13, 25]. Further research is needed on the effectiveness of comparing treatment effects on PROs using MAICs.

Another key challenge in SpA is the lack of reliable biomarkers of disease activity, structural damage and new bone formation [27]. Proxy outcomes representative of the underlying disease process, discomfort and/or disability are, therefore, frequently used in rheumatology clinical studies (e.g. disease activity outcome measures, such as the American College of Rheumatology (ACR) 20, are used as primary outcomes to discriminate the efficacy of newer drugs from placebo in RCTs, despite the fact that these parameters are not used clinically) [27, 28].

## Endpoints for MAICs in spondyloarthritis

Clinically meaningful hard endpoints are needed to demonstrate the additional value (e.g. improved disease status, long-term safety or reduced radiographic progression) of treatments for comparisons on the relative effectiveness to be made. Progression of joint damage is considered irreversible in SpA, and its prevention is an important treatment goal; this outcome can be assessed using imaging and a validated proxy scoring system, such as the modified Stoke Ankylosing Spondylitis Spinal Score (mSASSS) in AS and axial SpA [31], and by various scores of radiographic damage in hands and forefeet (e.g. the Sharp van der Heijde [SHS] method) in PsA [32]. While the mSASSS and the SHS are currently the most used measures of structural damage [33, 34], magnetic resonance imaging (MRI) has been proposed as a potentially more sensitive alternative [35]. Achieving remission, or low disease activity, which is the ultimate goal of SpA treatment [27], is also an example of clinically meaningful hard endpoints for head-to-head studies.

In contrast to the relatively rapid progression reported in rheumatoid arthritis [36], structural changes in axial SpA/AS generally take several years to progress. The shortest follow-up time in AS, based on the reliability and sensitivity to change of the mSASSS, is 2 years [37], while radiographic damage in PsA can be detected within 2 years of disease onset in almost half of patients [38]. The duration required to assess radiographic endpoints is, therefore, an important consideration when conducting comparative studies in SpA.

## Considerations for future head-to-head studies

Sample size estimations take into account the study design (superiority, equivalence, non-inferiority) and the study outcomes. Since more domains (axial and peripheral involvement, arthritis, enthesitis, dactylitis, psoriasis, uveitis, IBD etc.) have to be considered in SpA assessments, the identification of primary and secondary endpoints and sample size estimations are key issues when planning head-to-head studies. Whole-body MRI is a promising new technique for future clinical trials, as it allows assessment of both peripheral and axial joints and entheses [39].

Aside from traditional head-to-head studies, of which there are few published in SpA, a number of other comparative study designs exist, including trials comparing strategies instead of individual drugs and trials with an active control treatment arm. The TIght COntrol of Psoriatic Arthritis (TICOPA) trial—the first and only pragmatic RCT in PsA—compared intensive management versus standard care in early PsA [40]. The recently published SPIRIT P1 trial in PsA is an example of a placebo- and active-controlled clinical trial that compared two regimens of ixekizumab and an active adalimumab reference arm to treatment with placebo (note: the study was not powered to directly compare adalimumab and ixekizumab) [41].

## Conclusions

Indirect comparisons may be used to infer the relative effectiveness of different treatments in the absence of head-to-head data and can supplement the evidence from traditional sources. MAICs provide an important technique to control for differences in study design and population [13]; however, the greater the imbalance in the study design and/or population characteristics, the lower the actual sample size/precision of the analysis. Allowing academic researchers access to anonymised patient-level data from clinical trials may help improve the quality of MAICs as full data sets from each study could be compared. MAICs, unlike network meta-analyses, are of value for longer-term comparisons beyond the placebo-controlled phase of clinical trials; this is an important consideration for chronic diseases like AS and PsA whose management focuses on long-term treatment goals. Care should be taken when choosing endpoints to compare in longer-term extension arms, as some can be influenced by issues of unblinding and expectation bias owing to the lack of a concurrent placebo arm. Although MAICs have a number of limitations, including the exploratory nature of the analysis, they may be of particular value in generating hypotheses to inform the design of head-to-head studies. Despite these limitations, drug reimbursement agencies, such as the Australian Pharmaceutical Benefits Advisory Committee (PBAC), the UK NICE, the Scottish Medicines Consortium (SMC) and the Canadian Agency for Drug and Technologies in Health (CADTH), are increasingly accepting MAIC as a means of comparative effectiveness [42]. Improvements in the selection of clinical study endpoints and the general reporting of clinical trial data in SpA could greatly improve the quality of data to allow more informative indirect comparisons between the myriad of emerging therapies for SpA.

## Abbreviations

AS: Ankylosing spondylitis
ASDAS: Ankylosing Spondylitis Disease Activity Score
BASDAI: Bath Ankylosing Spondylitis Disease Activity Index
BASFI: Bath Ankylosing Spondylitis Functional Index
CADTH: Canadian Agency for Drug and Technologies in Health
CPDAI: Composite Psoriatic Arthritis Disease Activity Index
DAPSA: Disease Activity Index for Psoriatic Arthritis
DMARDs: Disease-modifying antirheumatic drugs
HAQ-DI: Health Assessment Questionnaire Disability Index
IBD: Inflammatory bowel disease
MAIC: Matching-adjusted indirect comparison
MDA: Minimal disease activity
MRI: Magnetic resonance imaging
mSASSS: Modified Stoke Ankylosing Spondylitis Spinal Score
NICE: National Institute for Health and Care Excellence
PASDAS: Psoriatic Arthritis Disease Activity Score
PBAC: Pharmaceutical Benefits Advisory Committee
PRO: Patient-reported outcome
PsA: Psoriatic arthritis
PsAQoL: Psoriatic Arthritis Quality of Life
RCT: Randomised controlled trial
SHS: Sharp van der Heijde score
SMC: Scottish Medicines Consortium
SpA: Spondyloarthritis
TICOPA: TIght COntrol of Psoriatic Arthritis
UK: United Kingdom
